# Embryonal Rhabdomyosarcoma of the Tongue in Adults

**DOI:** 10.3390/life13061255

**Published:** 2023-05-25

**Authors:** Alberto Díez-Montiel, Raúl Antúnez-Conde, Carlos Navarro Cuéllar, Manuel Tousidonis Rial, José Ignacio Salmerón, Nuria Bonsfills, Carolina Agra Pujol, Francisco Alijo Serrano, Santiago Ochandiano

**Affiliations:** 1 Department of Oral and Maxillofacial Surgery, Instituto de Investigación Sanitaria Gregorio Marañon (liSGM), Gregorio Marañon General University Hospital, 28007 Madrid, Spain; comunicacionrac@hotmail.es (R.A.-C.); cnavarrocuellar@gmail.com (C.N.C.); manuel@tousidonisrial.com (M.T.R.); jisalmeron@telefonica.net (J.I.S.); sochandiano@hotmail.com (S.O.); 2 ICIRE Institute for Reconstructive and Aesthetic Surgery, 28009 Madrid, Spain; nuria.bonsfills@gmail.com; 3 Department of Pathology, Gregorio Marañon General University Hospital, 28007 Madrid, Spain; caroagra@gmail.com (C.A.P.); pacoalijo@hotmail.com (F.A.S.)

**Keywords:** embryonal rhabdomyosarcoma, adult sarcoma, tongue neoplasm, head and neck cancer, case report

## Abstract

**Simple Summary:**

Rhabdomyosarcoma is the most common malignant soft tissue sarcoma in childhood and adolescence, with one-third of cases occurring in the head and neck region. A patient with a soft tissue mass on their tongue presented to our clinic. After an excisional biopsy, histology revealed an embryonal type of rhabdomyosarcoma, an extremely rare diagnosis in the adult population. An extension study was performed, followed by appropriate margin-free surgery with reconstruction using a buccinator flap and subsequent neoadjuvant chemotherapy. After 42 months, the patient remains free of disease. A thorough literature search for similar cases and treatments yielded only three comparable cases. The rarity of this pathology in adults necessitates the application of children’s treatment protocols, although the prognosis is worse than in childhood cases.

**Abstract:**

(1) Background: Rhabdomyosarcoma (RMS) is the most common soft tissue sarcoma in the first two decades of life. One third of cases appear in the head and neck, with 60% of these being embryonal type. RMS is extremely rare in adults, comprising only 1% of adult malignancies, and of those, only 3.3% are rhabdomyosarcomas. (2) Case report: A 46 y.o. male presented with a 1 cm exophytic pediculated painless lesion on the dorsum of his tongue, with progressive growth for 3 months. An excisional biopsy revealed an “embryonal rhabdomyosarcoma with fusocellular areas, with negative rearrangement for gen FOXO1A, negative MDM2 (only focal positivity), and positive INI-1”. Subsequent contrast-enhanced MRI concluded the presence of a lesion with imprecise margins in the right half-tongue, 15 × 8 × 7 mm (longitudinal × transverse × craniocaudal), compatible with a sarcoma. The patient underwent a partial centrolingual glossectomy followed by reconstruction with a buccinator muscle local flap. After surgery, he received chemotherapy with eight cycles of VAC (vincristine, actinomycin, and cyclophosphamide) protocol. The patient is now disease free after 42 months, with good tongue function. (3) Discussion and conclusions: Embryonal RMS is an extremely rare sarcoma in adults, and the location in the tongue is even more exceptional (only two more similar cases are reported in the literature). The prognosis in adults is significantly poorer than in children. A complete margin-free resection with an adequate chemotherapy protocol is the treatment of choice in cases such as these.

## 1. Introduction

Rhabdomyosarcoma (RMS) is a malignant soft tissue sarcoma arising from mesenchymal stem cells that differentiate towards striated muscle, with striated myoblasts in different differentiation stages [1,2,3,4]. According to the World Health Organization classification, it is a skeletal muscle tumor with origin in undifferentiated skeletal tissue [5]. RMS is the most frequent soft tissue sarcoma in children and adolescents, with an incidence of approximately 0.44 cases/100,000 people/year.

RMS is classified under the category of small round blue cell tumors. Typically, it shows positivity for specific muscular markers, including alfa-actyn, myosin, desmin, myoglobin, myogenin, and MYO-D [1,3,6,7]. In the fourth revision of the WHO Classification of Head and Neck Tumors, RMS can manifest as four histologic variants (embryonal, alveolar, pleomorphic, and spindle cells). The embryonal subtype is the most common, accounting for 60% of cases, and it is often located in the orbital region of children [6,8,9,10]. Accurate classification of RMS is important for staging, treatment, and prognosis. Clinical classification is based on “The Intergroup Rhabdomyosarcoma Study Clinical Grouping System” [11].

Loss of heterozygosity at the 11p15 locus is a characteristic of the embryonal RMS (ERMS), which is the location of the IFG-II gene. Additionally, more than 90% of cases show recurrent mutations affecting the receptor tyrosine kinase/RAS/PIK3CA, leading to overexpression of this gene with a growth factor role, thereby stimulating tumor cell growth. Other cytogenetic abnormalities that have been reported in ERMS include trisomy of chromosomes 2, 8, and 13.

Most RMS in children and adolescents is located in the head and neck. However, in adults, it is a rare tumor, that accounts for only 1% of all malignancies and 2–4% of all soft tissue sarcomas, which are more commonly found in the extremities. [1,2,3,4,5,6,8,9,10,12,13,14,15]. The head and neck RMS can be classified into three subtypes based on their location: parameningeal (nasal cavity, paranasal sinuses, mastoid area, and infratemporal fossa), non-parameningeal (oral cavity, oropharynx, face, cheek, parotid region, and soft tissues of the neck), and orbital. Parameningeal RMS has a worse prognosis due to the difficulty in completely removing the lesion and its proximity to intracranial space. Conversely, non-parameningeal and orbital RMS have a better prognosis [9]. Otherwise, The Inter-group Rhabdomyosarcoma Study Grouping System (IRS) classifies RMS patients into four clinical groups based on the histologic subtype, the presence or absence of lymph node metastasis, and the presence or absence of positive margins after surgical excision [11].

RMS typically metastasizes through hematogenous spread to the lungs, bone marrow, and brain, although lymphatic and contiguous spread are also possible. At diagnosis, approximately 60% of adults with RMS will have metastatic disease.

The best results for the treatment of RMS are obtained through the combination of surgery, adjuvant chemotherapy, and radiotherapy. Complete resection avoiding big or afunctional mutilation is the optimal first step. However, if that is not feasible, neoadjuvant chemotherapy and radiotherapy may be considered, with surgery delayed until afterward if possible. A variety of chemotherapy agents have been proven effective against RMS, including vincristine, dactinomycin, actinomycin D, doxorubicin, cyclophosphamide, iphosphamide, cisplatin, dacarbazine, etoposide, sodium phenylbutyrate, pazopanib, and everolimus. Additionally, RMS is highly sensitive to radiotherapy in children, with mean doses of 50 Gy (range 10–70 Gy) typically utilized [2,3,4,6,11,16].

Adults with RMS have a poorer prognosis compared to children. In the last four decades, there has been a significant improvement in survival rates among pediatric patients with RMS. While the 5-year survival rate in children has now surpassed 80%, in adults, it ranges from 27% to 40% in consecutive Intergroup Rhabdomyosarcoma Studies. Some authors have suggested that adult RMS is a distinct entity that requires different management than pediatric RMS [4,8,13,17]. Comparing adult and pediatric populations, adult RMS is associated with a higher incidence of metastatic recurrence. In addition, they do not respond as well as children to chemotherapy or radiotherapy, with worse tolerance to chemotherapy agents. In addition, some studies have reported a higher expression of multi-drug resistance proteins to those agents, higher than what is seen in children [3,18].

Head and neck sarcomas are only 1% of the malignancies in the oral cavity [19]. Furthermore, tumors located in the tongue are rare, and embryonal-type RMS in adults is extremely uncommon [20,21,22,23,24,25,26,27,28,29]. In this report, we present a rarity among rarities, an embryonal-type RMS in an adult tongue.

## 2. Case Description

Case Report. A 46 y.o. male patient was referred to the Oral and Maxillofacial Department of the Hospital Universitario Gregorio Marañón (Madrid, Spain) due to a progressively growing lesion at the center of the dorsum of the tongue that had been noticed 3 months prior. Upon clinical examination, an exophytic pedicled lesion was observed in the middle line of the dorsum of the tongue, measuring approximately 1 cm in diameter, with an elastofibrotic consistency, painless, non-friable, and with no active bleeding or satellite lesions. Tongue mobility was normal, and no palpable pathological lymph nodes were detected. The thoracic and abdominal physical exams were unremarkable. A chronologically ordered sequence of events (timeline) is presented in Table 1.

Diagnostic assessment. An excisional biopsy was carried out under local anesthesia. It revealed an ulcerated lingual mucosa and granulation tissue exhibiting superficial and deep mesenchymal proliferation. The findings were consistent with embryonal rhabdomyosarcoma. The sample showed negative rearrangement for FOXO1A gen, negative although with positive focality for MDM2 gen, and positive for INI-1 gen.

**Figure 1:**
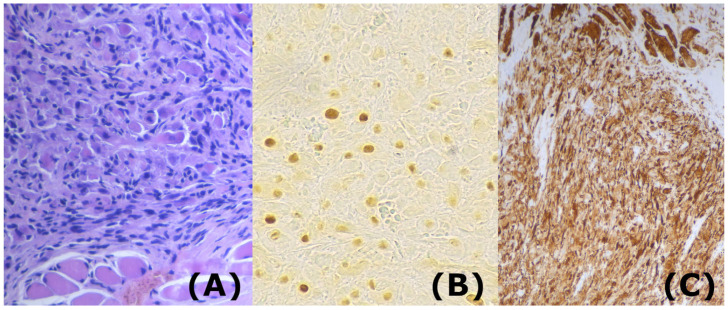
Histopathology of the resection sample. A: Hematoxilin–Eosin (X40). The high-power field shows numerous rhabdomyoblasts with brightly eosinophilic cytoplasm. (**A**) Compact area with rhabdomyoblastic differentiation adjacent to an area with some atypical and hyperchromatic cells growing between normal muscle fibers. (**B**): Immunohistochemistry (X20). Many tumor cells show strong nuclear immunostaining for Myogenin. (**C**): Immunohistochemistry (X20). The tumoral cells express diffuse strong positivity for Desmin. Genomic studies identified somatic driver mutations involving the RAS pathway (NRAS, KRAS, HRAS, NF1, and FGFR4) involving effector fPIK3K (PTEN and PIK3CA).

Histopathological analysis of the excised mass revealed a highly cellular tumor composed of small–medium-sized undifferentiated cells with atypical and hyperchromatic, frequently nucleolated nuclei and indistinct cytoplasm (Figure 1). Immunohistochemical evaluation revealed positivity for desmin, smooth muscle antigen HHF35, and myogenin, with a Ki-67 proliferation index of 65%. The diagnosis was based on the presence of primitive and spindle cell morphology with scattered differentiated rhabdomyoblasts, as well as the expression of desmin and heterogeneous nuclear staining for myogenin and/or MYOD1. The neoplastic cells lacked FOXO1 gene fusion, and this condition distinguishes ERMS from solid ARMS (alveolar rhabdomyosarcoma). They were also negative although with positive focality for MDM2 gen and positive for INI-1 gen. Thus, based on the histological and immunohistochemical findings, the diagnosis was embryonal rhabdomyosarcoma with fusocellular areas. 

Following the excisional biopsy, a CT scan was performed, which revealed a cavity of 12 × 6 × 10 mm (transverse × sagittal × longitudinal) in the right paramedian region of the tongue. The cavity was not related to the lingual extrinsic muscles, and it was suspected to be a postsurgical cavity. Subsequently, MRI of the neck was conducted, which showed a lesion in the dorsal area of the middle third of the right half-tongue. The lesion measured 15 × 8 × 7 mm (anterior–posterior, transverse, and craniocaudal) and demonstrated enhancement with IV contrast dye. Although the margins of the lesion were undefined, it did not invade the midline. The MRI report identified the lesion as a sarcoma located in the dorsal area of the right half-tongue.

**Figure 2:**
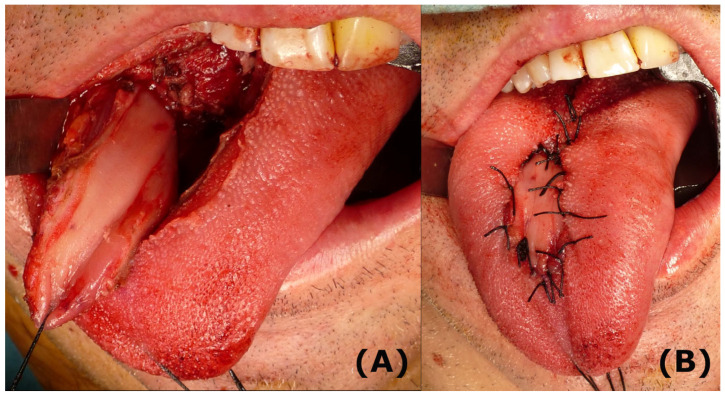
Surgical pictures from the partial glossectomy and the local buccinator flap. (**A**): The defect is clearly seen, and the flap is presented in the defect. (**B**). Final result.

Treatment. After MRI, the patient underwent a centrolingual partial glossectomy with reconstruction of the local buccinator muscle flap. The procedure was performed without any postoperative complications (Figure 2). 

Six weeks after the surgery, once the patient had healed properly, chemotherapy with the VAC protocol (vincristine, Adriamycin, and cyclophosphamide) was initiated. The patient received eight cycles of the protocol, although the last four cycles were administered without cyclophosphamide due to toxicity.

Follow-up. Our case is disease-free 42 months after surgery. The patient was comfortable with the outcome and did not present any observable masses, issues with tongue mobility, or feeding problems.

## 3. Discussion

To the best of our knowledge, this paper represents the most comprehensive review of adult tongue rhabdomyosarcoma (RMS) to date. We conducted a thorough search across multiple databases to identify the relevant studies, which proved challenging given the rarity of this pathology. Despite our efforts, some cases may have been missed due to incomplete reporting of patient age, tumor type, and/or location in certain series. Given the scarcity of adult tongue RMS cases, standardization and systematic review of the literature remains difficult if not impossible.

A comprehensive search was conducted in four databases (PubMed, Scopus, EMBASE, and Google Scholar) using the keywords “rhabdomyosarcoma” in combination with “head and neck”, “embryonal”, “adult”, and “tongue”. A total of 113 papers were identified, including 37 case reports and 67 series, with 9 reviews reporting on nearly 15,000 cases. Among the series, there were both short [1,7,30] and large studies, with the latter usually sourced from multicenter survival registries [31,32,33]. After filtering for adult cases, the number of papers was reduced to 60 (19 case reports and 41 series), which was further reduced to 53 (16 case reports and 37 series) when considering adult age and embryonal subtype.

In many of the published articles, the location of the tumor in the tongue was not specified. When examined, among the 113 papers, only 67 cases were located in the tongue. When adult age was considered, the number of cases in the tongue was 49, and this decreased to 29 when the embryonal type was added in 8 publications. Three publications did not clarify if the tumor of the tongue was from an adult or a child [27,28,29]. Thus, only three cases in four papers matched the characteristics of the case presented in this paper: one case report by Baldi et al. [21], one case report from the series by Doval [22], that was the same case found in the series by Pavithran [26], and one case report by Bozorg-Grayeli [20]. The first one showed a 65 y.o. male, treated with surgery and radiotherapy, who was disease-free at the end of a 48-month follow-up [21]. The second one reported a 32 y.o. male, treated with only chemotherapy and radiotherapy, who died 24 months after treatment [26]. The third case, described by Bozorg-Grayeli, was incomplete because only the abstract could be retrieved [20], and it did not mention the age or follow-up. (Table 2).

RMS is the most frequently occurring sarcoma in the pediatric population [17]. Despite this, adult RMS has not been extensively studied, and the published data are primarily derived from case series in single facilities, with no survival analysis. Statistic descriptors from retrospective series show a poor prognosis, significantly lower than the 5-year 80–84% rates observed in children. The most common symptom of RMS is the presence of painful swelling, with a slightly higher incidence among males compared to females (1.5:1) [17].

Age is one of the most important prognostic factors in RMS. Children older than 10 y.o. also show worse failure-free survival rates than children under that age. Additionally, the definition of an “adult” patient can vary between studies (over 15 y.o., over 18 y.o., or over 21 y.o., among others), potentially introducing bias when comparing adult and pediatric populations. Positive prognostic factors (both for adults and children) include young age, low-risk group, favorable histology, favorable primary site (non-parameningeal), non-metastatic disease, good response to chemotherapy and/or radiotherapy, small tumor size, and negative margin status after primary resection [4]. Negative prognostic factors in children include older age, alveolar subtype, and regional extension of the disease. Sultan et al., in a large series on 2600 patients, confirmed those negative predictors in adults except for the alveolar subtype and the unfavorable primary site [31]. Almost 45% of adult patients will develop distant metastasis, most commonly in the lungs [6,9,18]. The most common cause of death is tumoral growth and the invasion of vital deep structures.

Due to the rarity of ERMS in adults, there is no consensus on the optimal treatment approach. Based on the current knowledge, the best therapeutic strategy may involve a combination of surgery, radiotherapy, and chemotherapy. However, wide resection surgery is not always feasible, particularly in head and neck patients, due to the risk of significant mutilation. Therefore, neoadjuvant chemotherapy followed by radiotherapy may be a viable alternative, even for microscopic metastatic disease. Given the high incidence of lymph node metastasis, radiotherapy to the neck should be considered in adult RMS cases.

If adult ERMS is infrequent, the location in the tongue seems exceptional. Differential diagnosis should be made with other malignant lesions such as malignant peripheral nerve sheath tumors with heterologous rhabdomyosarcomatous differentiation (known as a “malignant Triton tumor”), especially in patients with neurofibromatosis type I (NF1). Sarcomatoid carcinoma, originating in the skin, mucosa, or salivary gland, may also be a possibility, particularly in older patients who have previously undergone radiation. Additionally, small round cell tumors such as Ewing’s sarcoma, small cell melanoma, lymphoblastic lymphoma, neuroblastoma, primitive neuroectodermal tumors, desmoplastic small round cells tumors, or chordocarcinoma should also be considered [1,7].

Prognosis, both in children and adults, is different in “favorable” (orbit, non-parameningeal head and neck, non-bladder/non-prostate genitourinary tract, and non-biliary tract) and “unfavorable” locations, although the difference is smaller in adults than in children [4,31,34]. The tongue is one of the theoretical, but infrequent, favorable sites. In those sites, the response to chemotherapy was better [35]. Otherwise, the extraordinary location of RMS in the tongue and the scarce literature about it made our multidisciplinary sarcoma committee plan the treatment without a standardized protocol. If the protocols for children seemed effective for adults, head and neck guidelines were considered in this case, with more surgical resection and less added chemotherapy and radiotherapy [36,37].

Our case is a perfect match for the exceptional findings of RMS: a mid-aged adult, embryonal type, and located in the tongue. Management of the tumor was performed in a pediatric-like way, with adjuvant chemotherapy. A systematic review of the pathology seems challenging because of the rarity, but also because studies on larger series, on IGRS registries, may include cases from short series or case reports, and the specific features of the cases are not always defined. The two published cases that match with ours regarding subtype, age, and location, are shown in Table 2. Due to the difficulty of diagnosis, controversy, and exceptional nature of these cases, protocolized management for ERMS of the tongue in adults could not be identified.

## Figures and Tables

**Table 1:** Timeline of the case report.

Timeline	Event	Results
March 2019	The appearance of a growing painless mass on the tongue	Attention in primary care
April 2019	Attention in primary care	Exophytic pedunculated lesion, 1 cm diameter, in the midline of the dorsum of the tongue Urgent referral to the Department of Oral and Maxillofacial Surgery
25 April 2019	Department of Oral and Maxillofacial Surgery. Excisional biopsy performed	Embryonal rhabdomyosarcoma with fusocellular areas
22 May 2019	Excisional biopsy of the dorsum of the tongue	Scar tissue
23 May 2019	CT of the neck	A cavity found around the excisional biopsy. Inconclusive data about tumoral remains
6 June 2019	Contrast-enhanced MRI	1.5 × 0.8 cm lesion on the dorsum of the right hemi-tongue, possible sarcoma
24 June 2019	First visit to the Medical Oncology/Sarcoma Committee.	Decision: surgery + adjuvant chemotherapy
9 July 2019	Central partial glossectomy + reconstruction with buccinator flap. Mass and samples of margins obtained	No events. Margins without tumor cells
18 July 2019	Definitive results of pathology	Scar fibrosis and inflammatory reaction with giant cells, with no evidence of tumor cells
July 2019	Extension study performed: bone scan, thorax simple X-ray, and PET-CT	Extension study negative
August 2019–January 2020	Chemotherapy (VAC, eight cycles, the first four with cyclophosphamide)	No events
January 2020–July 2022	Follow-up	No signs of local recurrence or metastatic disease

**Table 2:** Previous cases of RMS of the tongue in adults. E = embryonal. NS = not specified.

Author and Year	N	Age	Gender	Type of RMS	Treatment	Survival
Bozorg-Grayeli 1993 [20]	1	NS	Male	E	Surgery + radiotherapy	NS
Doval 1994 [22], Pavithran 1997 [26]	1	32	Male	E	Chemotherapy + radiotherapy	Death 24 months after
Baldi 2004 [21]	1	65	Male	E	Surgery + radiotherapy	Disease-free 48 months after

## Data Availability

The data presented in this study are available from the corresponding author upon request.
